# Buffalo Medical and Surgical Association

**Published:** 1891-08

**Authors:** W. S. Renner

**Affiliations:** Secretary


### BUFFALO MEDICAL AND SURGICAL ASSOCIATION.

Reported by W. S. RENNER, M. D., Secretary.

REGULAR meeting held Tuesday, June 9, 1891, in the parlors of the Hotel Iroquois, at 8.30 P. M.

THE PRESIDENT, Dr. WM. C. PHELPS, in the chair.

Dr. WM. C. KRAUSS read a paper, entitled, The People vs. Sadie McMullen — A Medico-Legal Case.

The essayist reviewed closely the family history, revealing a terrible state of things. Alcoholism, mania, dementia, etc., are of frequent occurrence, going back as far as the great-grandfather. Of twenty-five members of the family, thirteen are mentally deranged. An important point is the relation existing between the grand-parents on the father's side — being first-cousins. The early history of Sadie McMullen was then taken up, the first appearance of epilepsy when two years old, caused by fright, their changing into epileptoid as a result of treatment, their frequent occurrence, particularly at or near her menstrual periods, were all carefully reviewed. The last visit to Akron, the two letters which she received from Buffalo, and the crime, are familiar to all. On examining the prisoner at the jail in company with Drs. Mickle and Persons, nothing could be detected showing hysteria or permanent insanity. The theory of the defense was that the crime was committed while the prisoner was in an epileptoid state,— and hence irresponsible.

### DISCUSSION.

Dr. WETMORE had seen two or three cases of epilepsy in young girls, probably due to hereditary influences.

Dr. COAKLEY related the history of an epileptic between fifty and sixty years of age, who walked, while unconscious, off the rear of a moving passenger train, but was not injured.

Dr. HAYD said that the paper suggested the possibility of many of our criminals being in an epileptoid state when they committed crimes.

Dr. THORNER related the case of a gentleman who had wandered about from January 14th to May 14th, in various parts of the United States. He had no idea how he had gotten from place to place, where he had been, or how he had come into possession of various articles which he had on his person.

Dr. RING said that it seemed peculiar that such people could move about without being apprehended.

Dr. GROSVENOR thinks that we can look to alcoholism and the ancestors for the production of nerve-degeneration and defective mentality in Sadie McMullen. He enumerated many conditions which can be attributed to alcoholism in the ancestors. He thought that if chronic inebriates were cared for by the state, transmission of such tendencies might be prevented.

Dr. JONES related the history of a case of classic epilepsy ; this gentleman went about in a dazed condition for days — such people were often supposed to be drugged. He objected to the naming of the epileptiod state "double consciousness," as no person could be in two conditions of mind at one time.

Dr. STOCKTON mentioned an epileptic, twenty-five years old, with good family history ; the condition was brought on by fright when the patient was seven years old. He had had classic epilepsy ; had been confined in an asylum while insane ; has now *petit mal.* Often walks two or three blocks perfectly naturally, although not conscious of what he is doing ; at other times he stands in some fixed attitude (often ridiculous). He spoke of epilepsy in a patient of his associated with early closure of the fontanelles.

Dr. HAYD asked if this epileptoid condition were always associated with, or followed, *petit mal*, and if it ever developed independently of *petit mal.*

Dr. PHELPS thought that physicians should advise patients with *grand mal* not to put themselves in dangerous places. He had had a patient who had fallen into a soap kettle after he had advised him to discontinue his occupation as a soap-maker ; this patient was scalded to the umbilicus. Speaking of marriages of consanguinity, he related the history of a family of five children, whose parents were first-cousins; of these, one had hare-lip, one chorea, one is in the Providence Asylum, one is in the State Hospital, while the fifth is a very peculiar person. He stated that he was called in by the District Attorney to examine the case of Sadie McMullen. He found family history and physical examination same as Dr. Krauss had related ; when he examined her in jail she did not appear other than sane, and she told Dr. Phelps that she had been told by her attorney not to say anything to any person without his permission ; neither would she answer any questions until the doctor obtained permission for her to do so from her attorney. He found that she was subject to attacks of wandering off, but could not get the history of many. Three things were to be noted in her attempt to drown herself ; the road where there was but little water ; she helped herself out of the water, and did not try to get back into the water after she was taken out. He considered the case one of hysteria ; he did not think she was conscious from the time she left the store until she entered the water ; he did not consider her accountable for her acts ; he knew nothing of her history since her confinement in the State Hospital ; legally it amounted to the same thing whether she were suffering from epilepsy or hysteria.

Dr. Krauss said that at present she is gaining in every respect, that Drs. Andrews, Hurd, and others of the staff at the State Hospital, hold that she never had a homicidal tendency in the world. In reply to Dr. Hayd, he said the epileptoid state does not come on suddenly ; you must have a previous history of epilepsy ; it follows either *grand mal* or *petit mal* ; he agreed with Dr. Jones that there is no such thing as "double consciousness," and had avoided the use of this name in his paper.

Meeting adjourned at 10.30 P. M.

